# Ethnicity Recording and Equity of Access in Child and Adolescent Mental Health Services: A Re-Audit of East Lancashire CAMHS

**DOI:** 10.1192/bjo.2026.11169

**Published:** 2026-06-30

**Authors:** Meghna Nair, Kavitha Ramamurthy, Venugopal Duddu

**Affiliations:** 1 University of Leeds, Leeds, United Kingdom; 2 Children and Young People’s Mental Health services (CYPMH), Burnley, United Kingdom; 3 East Lancashire and South Cumbria Foundation NHS Trust (LSCFT), Burnley, United Kingdom

## Abstract

**Aims:**

Ethnic minority children and adolescents are consistently under-represented in UK mental health services. Accurate ethnicity recording is essential for monitoring equity of access and informing service improvement. A baseline clinical audit conducted in 2020 across East Lancashire Child and Adolescent Mental Health Services (CAMHS) identified incomplete ethnicity recording and significant under-representation of Asian service users relative to the local population.

This re-audit reassessed the original audit standards by evaluating ethnicity recording completeness and whether the ethnic composition of CAMHS service users reflected the local under-18 population. It tested the hypotheses that ethnicity recording completeness would improve following the 2020 audit and that service use would reflect local population demographics.

**Methods:**

A re-audit was conducted using electronic health records for all open CAMHS cases (n=1626) across East Lancashire Child and Adolescent Services as of 6 May 2025. Analyses were undertaken at locality level (Blackburn with Darwen; Burnley and Pendle; Hyndburn, Ribble Valley and Rossendale). Descriptive statistics were used to assess ethnicity recording completeness. Population-based access rates per 1,000 children were calculated using 2011 Census under-18 population denominators, and observed and expected numbers of Asian CAMHS service users were compared using chi-square goodness-of-fit tests.

**Results:**

Ethnicity recording completeness improved overall between 2020 and 2025, increasing from approximately 78% at baseline to 88% at re-audit, with improvement seen in most localities. Despite this, Asian children and adolescents remained significantly under-represented among CAMHS service users. Access rates for Asian children were consistently lower than those for non-Asian children across all localities (approximately 2–5 vs 12–18 per 1,000 children, respectively), and observed numbers were significantly lower than expected based on population proportions (χ² goodness-of-fit, p < 0.001).

**Conclusion:**

This re-audit demonstrates measurable improvement in ethnicity recording completeness within East Lancashire CAMHS since the 2020 baseline audit, indicating partial completion of the audit cycle. However, improved data quality has not translated into equitable access to CAMHS, with Asian children remaining under-represented and access rates persistently lower than expected.

